# A Neuro-Informatics Pipeline for Cerebrovascular Disease: Research Registry Development

**DOI:** 10.2196/40639

**Published:** 2023-07-21

**Authors:** Thomas B H Potter, Sharmila Pratap, Juan Carlos Nicolas, Osman S Khan, Alan P Pan, Abdulaziz T Bako, Enshuo Hsu, Carnayla Johnson, Imory N Jefferson, Sofiat K Adegbindin, Eman Baig, Hannah R Kelly, Stephen L Jones, Gavin W Britz, Jonika Tannous, Farhaan S Vahidy

**Affiliations:** 1 Department of Neurosurgery Houston Methodist Houston, TX United States; 2 Center for Health Data Science and Analytics Houston Methodist Houston, TX United States; 3 Weill Cornell Medicine New York, NY United States; 4 Neurological Institute Houston Methodist Houston, TX United States

**Keywords:** clinical outcome, intracerebral hemorrhage, acute ischemic stroke, transient ischemic attack, subarachnoid hemorrhage, cerebral amyloid angiopathy, learning health system, electronic health record, data curation, database

## Abstract

**Background:**

Although stroke is well recognized as a critical disease, treatment options are often limited. Inpatient stroke encounters carry critical information regarding the mechanisms of stroke and patient outcomes; however, these data are typically formatted to support administrative functions instead of research. To support improvements in the care of patients with stroke, a substantive research data platform is needed.

**Objective:**

To advance a stroke-oriented learning health care system, we sought to establish a comprehensive research repository of stroke data using the Houston Methodist electronic health record (EHR) system.

**Methods:**

Dedicated processes were developed to import EHR data of patients with primary acute ischemic stroke, intracerebral hemorrhage (ICH), transient ischemic attack, and subarachnoid hemorrhage under a review board–approved protocol. Relevant patients were identified from discharge diagnosis codes and assigned registry patient identification numbers. For identified patients, extract, transform, and load processes imported EHR data of primary cerebrovascular disease admissions and available data from any previous or subsequent admissions. Data were loaded into patient-focused SQL objects to enable cross-sectional and longitudinal analyses. Primary data domains (admission details, comorbidities, laboratory data, medications, imaging data, and discharge characteristics) were loaded into separate relational tables unified by patient and encounter identification numbers. Computed tomography, magnetic resonance, and angiography images were retrieved. Imaging data from patients with ICH were assessed for hemorrhage characteristics and cerebral small vessel disease markers. Patient information needed to interface with other local and national databases was retained. Prospective patient outreach was established, with patients contacted via telephone to assess functional outcomes 30, 90, 180, and 365 days after discharge. Dashboards were constructed to provide investigators with data summaries to support access.

**Results:**

The Registry of Neurological Endpoint Assessments among Patients with Ischemic and Hemorrhagic Stroke (REINAH) database was constructed as a series of relational category-specific SQL objects. Encounter summaries and dashboards were constructed to draw from these objects, providing visual data summaries for investigators seeking to build studies based on REINAH data. As of June 2022, the database contains 18,061 total patients, including 1809 (10.02%) with ICH, 13,444 (74.43%) with acute ischemic stroke, 1221 (6.76%) with subarachnoid hemorrhage, and 3165 (17.52%) with transient ischemic attack. Depending on the cohort, imaging data from computed tomography are available for 85.83% (1048/1221) to 98.4% (1780/1809) of patients, with magnetic resonance imaging available for 27.85% (340/1221) to 85.54% (11,500/13,444) of patients. Outcome assessment has successfully contacted 56.1% (240/428) of patients after ICH, with 71.3% (171/240) of responders providing consent for assessment. Responders reported a median modified Rankin Scale score of 3 at 90 days after discharge.

**Conclusions:**

A highly curated and clinically focused research platform for stroke data will establish a foundation for future research that may fundamentally improve poststroke patient care and outcomes.

## Introduction

### Background

Modern-day health care is at the precipice of a digital revolution. Driven by technological advancements and legislative considerations, health care systems have made massive shifts toward electronic health record (EHR) systems that capture both structured and unstructured data streams [1]. The large-scale digitization of health information, coupled with an estimated 36 million annual hospitalizations (before the COVID-19 pandemic) [2], fuels the rapid generation of data on physiological and pathological human states both within and outside health care institutions. Over the past decade, leading health care organizations have made substantial investments toward optimizing health care “big data” to improve the quality of care, enhance patient-centered outcomes and experiences, reduce waste, and understand targets for personalized medicine. Although advancements have been made, the health care data *asset-to-utilization* ratio remains low [3]. There is a growing recognition of the critical need to establish learning health care systems (LHSs) within and across health care organizations, a need that has been exponentially exacerbated during the COVID-19 pandemic [4,5]. Conceptually, LHSs enable hospitals to function as active learning environments, where evidence synthesis from real-world data is a by-product of clinical care [6]. The evidence is incorporated into clinical care and hospital operations, leading to more targeted and effective treatment [7] through processes that are continually evaluated for effectiveness and efficiency. In principle, this iterative cycle negates a notoriously long lag between traditional research and implementation cycles while also ensuring that evidence is contextually pertinent and readily actionable. Establishing an LHS is not trivial; however, it requires overcoming major technological and behavioral challenges [8,9].

In addition to the traditional complexities in the assimilation and analyses of big data (volume, velocity, and variety), a fundamental challenge is a lack of optimization within current EHR systems. More specifically, EHR data structures facilitate administrative functions, revenue generation, and hospital encounter tracking rather than providing clinically meaningful, longitudinally coherent, and patient outcome–centered data models for specific disease domains [10]. Moreover, digital management systems for various unstructured data streams, such as imaging, clinician notes, and physiological computation (waveform) data, are often poorly integrated, and pulling from these diverse clinical data sources may bias the resulting data structures when data teams do not effectively interface with clinicians and data providers, handicapping effective LHS progress [11,12]. Therefore, dedicated efforts by a diverse team of experts are needed to assemble and curate pipelines of validated and actionable evidence around major clinical domains [10]. These teams must be directly integrated with care teams and researchers to avoid bias as EHR data transition from the care zone to the database and research zones [9,10].

Cerebrovascular disease (CVD) and stroke are a prime clinical domain to benefit from dedicated research data structures. The risk factors and burden of CVD are on the rise, with poor outcomes, including disability, cognitive impairment, and death, disproportionately affecting underserved and marginalized populations [13]. This severity is compounded by the limited number of treatment options and further disparities in access to care [14]. A comprehensive prehospital, hospital, and posthospital care spectrum is required to reduce stroke burden and improve outcomes, yet the area has seen few direct LHS developments, with most implementations focusing primarily on oncology and neurology [7,15]. It is imperative that a health care system be established to learn from this critical and growing patient population and harmonize data across all possible internal and external resources to produce clinically meaningful and actionable insights.

### Objectives

We provided the rationale, context, repository structure, import pipelines, and governance of an LHS model for populations and patients with CVD and stroke across an 8-hospital tertiary health care system with an expansive population health and emergency care network in the greater Houston metropolitan area, one of the United States’ most diverse populations. This created resource, the Registry of Neurological Endpoint Assessments among Patients with Ischemic and Hemorrhagic Stroke (REINAH), was constructed using a series of specialized extract, transform, and load (ETL) data pipelines that effectively convert EHR data into an analyzable format, with oversight from clinical professionals and practiced epidemiologists. The resulting database is easily accessible for minimum-risk observational research and contains rich data that can be interwoven with other internal and external resources. In essence, we established a rich data platform that can support future projects and artificial intelligence initiatives to improve the detection, treatment, and overall outcomes of patients with stroke.

## Methods

### Rationale for Repository Establishment

The hospital environment is becoming increasingly technologically integrated, with information generated by hospital operations flowing into institutional EHRs and the picture archiving and communication systems (PACSs). This constant growth creates big data sets that can be harnessed for clinical research, machine learning, and artificial intelligence initiatives. Effective LHSs fundamentally seek this development, incorporating and analyzing internal and external data sources to provide improved efficiency and efficacy. Although the LHS model should ideally be sought in all cases to serve the collective good, it is perhaps most critical in cases where treatment options are limited and disparities are evident. To provide a fertile research environment and further the mission of Houston Methodist (HM) Hospital as an LHS entity, we sought to develop the REINAH repository for patients with stroke. Initially designed as a comprehensive system for investigating the impacts and outcomes of intracerebral hemorrhage (ICH), REINAH has grown to incorporate all types of stroke and affiliated CVD pathologies.

### Setting

#### Overview

The HM hospital system comprises 8 hospitals and 14 emergency care centers across the Houston Metropolitan Statistical Area (HMSA), including a main campus, an academic institute within the Texas Medical Center (TMC), and 6 community hospitals. The HMSA is the fifth-most populated statistical area in the United States, with a population of approximately 7.2 million [16]. The population of the HMSA is highly diverse, consisting of 7.8% Asian, 16.8% Black or African American, 38% Hispanic, 35.1% non-Hispanic White, and 2.3% other racial or ethnic subgroups [16]. To best serve this heterogeneous population, HM has established an accountable care organization in contract with the Centers for Medicare and Medicaid Services (CMS). This infrastructure has improved care quality and financial savings both before and during the COVID-19 pandemic, earning a CMS quality score of 99.5% [17].

As part of its expansive care network, HM includes 7 certified stroke centers available to assess and treat transient ischemic attack (TIA), acute ischemic stroke (AIS), ICH, and subarachnoid hemorrhage (SAH). Comprehensive stroke centers at HM offer interdisciplinary rapid-response systems with an affiliated stroke outpatient clinic for continued care during recovery. Patients admitted to the intensive care unit (ICU) are continuously monitored using the virtual ICU (VICU) system, which provides real-time measurement of physiological signals, including electrocardiograms (ECGs); blood pressure; oxygen saturation; respiration; and, when possible, electroencephalography. As part of the Sepsis Early Recognition and Response Initiative (SERRI), patients are also frequently monitored for markers of sepsis, septic inflammatory response syndrome, and delirium by trained professionals [18]. This provides a robust, data-rich environment for the assessment and care of patients with stroke. The direct patient care environment is supplemented by 13 diagnostic imaging and radiology centers located around the greater Houston area offering computed tomography (CT) and magnetic resonance imaging (MRI) services [19]. In addition, the HM Translational Imaging Center, located at the primary TMC location, features a 7-tesla MRI scanner that has been approved for clinical use (Siemens 7T MEGATOM Terra).

#### HM EHR System

Patient encounters and interactions at HM are recorded using an EHR system (Epic Systems Corporation), which spans May 2016 to the present day. Records generated at any of the HM constituent hospitals are standardized and synchronized across the system, thereby maintaining patient encounter continuity within EHRs.

The information stored within the EHR includes patient- and encounter-level factors. Patient information recorded from EHRs is largely demographic, including medical record number (MRN), gender, ethnicity, current level of education, current address and zip code, and current marital status. Encounter-level factors include all information related to specific incidences of patient contact, including inpatient, outpatient, and billing encounters. Associated data points include age at admission; admission characteristics; diagnoses and comorbidities; administered and reported medications; imaging; procedures; discharge characteristics; and flow sheet, vital, and laboratory values (eg, hematocrit, blood glucose, and prothrombin time). Patient and encounter information is primarily linked via the MRN, which remains consistent across all HM locations and interactions, creating longitudinal data sets for every individual.

Although this EHR structure contains a wealth of patient information, its native format is primarily optimized for administrative and billing processes. To transform this into a more clinically relevant format, a relational SQL database was established. Supported by a team of experienced data scientists and EHR researchers, a process was established to populate the database with clinical information from patients diagnosed with CVD.

### REINAH Structure and EHR Data Pipeline

#### Patient Inclusion

The REINAH registry collects and cleans data from all patients with a primary encounter or history of CVD. Patients are included if they received either primary admission or discharge International Classification of Diseases, 10th revision (ICD-10), codes I60 (SAH), I61 (nontraumatic ICH), I62 (nontraumatic intracranial hemorrhage), I63 (AIS), S06 (traumatic ICH), G45 (TIA), or cerebral amyloid angiopathy (CAA; ICD-10 code I68.0). Patients with records indicating a history of CVD, identified through the same ICD-10 criteria, are similarly included to maintain continuity for patients after stroke. Patients are excluded if imaging does not show CVD consistent with the diagnostic code or if they request that their data be excluded from the study. The lists of included patients are generated on a weekly basis, and patient data are imported via a refined, 2-level ETL process.

#### ETL Processes

Briefly, ETL refers to the process of ingesting data from disparate sources and staging them in a central repository in the form of an SQL server database. Data from the EHR are first cleansed, given a standard format, and denormalized to make them compatible for efficient report building. The data definitions and business rules for these sets are implemented through SQL programming, which imports and organizes data into separate category-specific (eg, comorbidities, laboratory results, and admission characteristics) tables and views within the database.

After patients are identified, data are retrieved for their primary CVD encounter. This is enriched by adding events surrounding primary stroke encounters, such as patient vitals, laboratory test results, procedures, medication, and imaging information from the EHR sorted into their respective tables of interest. Missing data and data with erroneous characteristics (eg, blood pressure records with higher diastolic than systolic pressures and unrealistic date information) are encoded as “NULL” values that can be addressed by data analysts and researchers on a project-specific basis.

**Figure 1:**
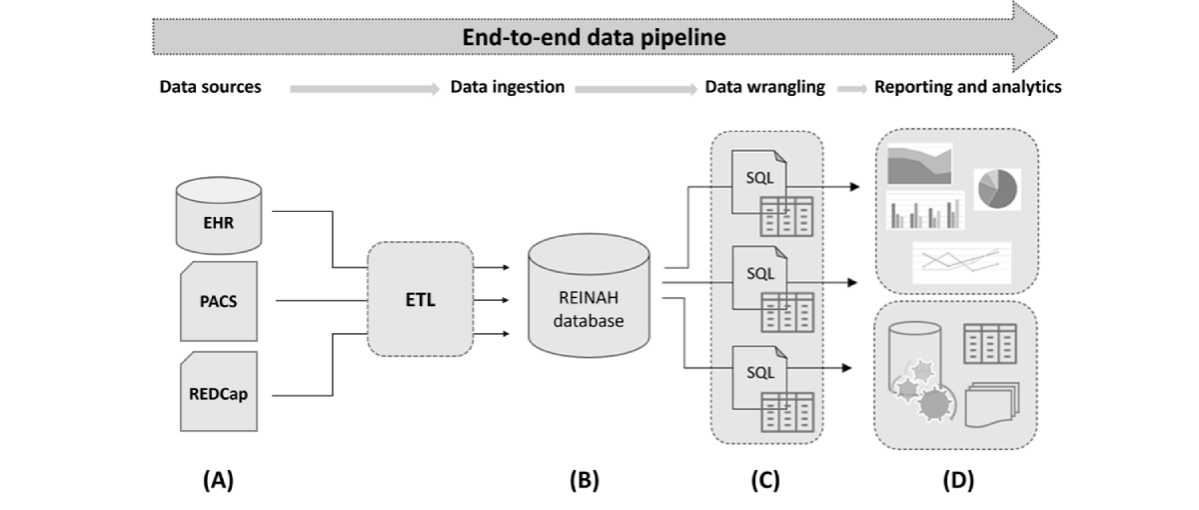
End-to-end automated data feed. (A) Inbound extract, transform, and load (ETL) feeds from Epic (electronic health record [EHR]), picture archiving and communication system (PACS) imaging, and REDCap (Research Electronic Data Capture) survey data. (B) Central data repository for staging ingested data. (C) SQL procedures defining measures related to stroke population. (D) Advanced analytics for data science, natural language processing, interactive dashboards, and reporting.

**Figure 2:**
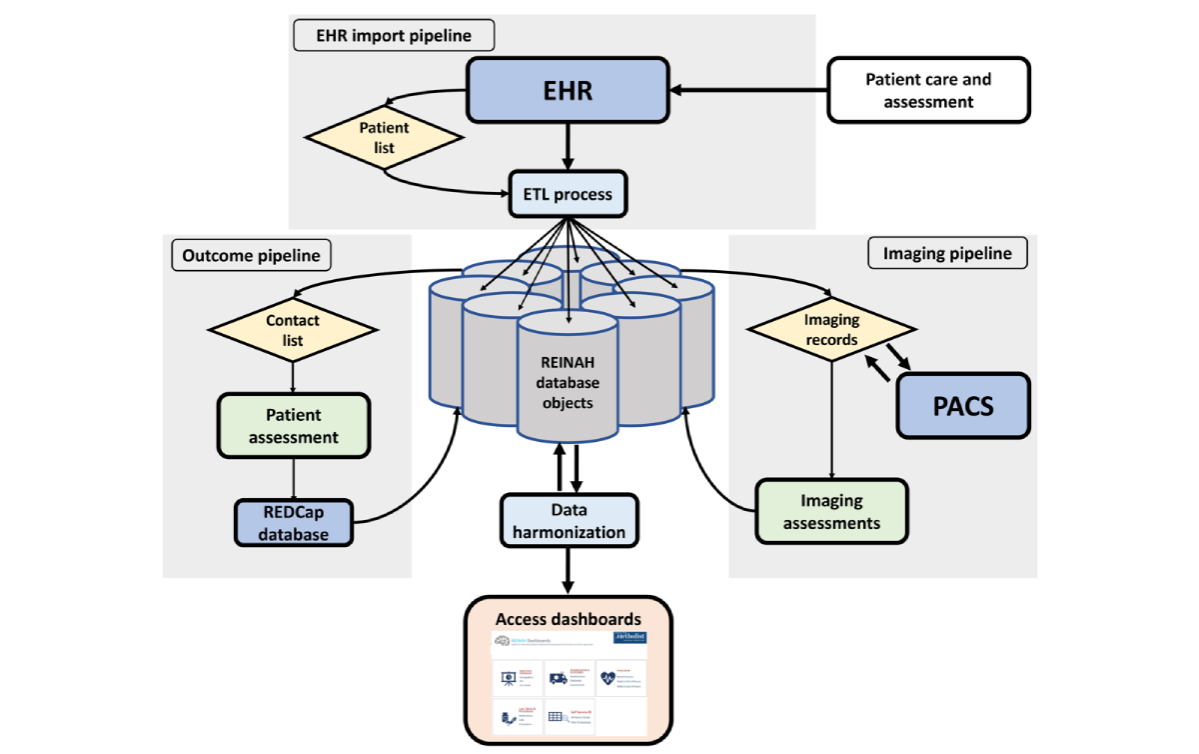
A flow sheet of the Registry of Neurological Endpoint Assessments among Patients with Ischemic and Hemorrhagic Stroke (REINAH) structure and data elements. External resources are depicted in dark blue, with bridging steps in light blue. Records for patient and data selection are shown in yellow, with assessment methods in green. Finally, front-end access is shown in pink. EHR: electronic health record; ETL; extract, transform, and load; PACS: picture archiving and communication system; REDCap: Research Electronic Data Capture.

Information on imaging procedures relevant to stroke occurrences—CT, MRI, angiography from both CT and MRI, positron emission tomography, single–photon emission CT, x-ray, ultrasound images, and patient waveforms—is retrieved and sorted into imaging database tables. Paired imaging files are identified via EHR-imported accession numbers, anonymized, and retrieved from the institutional PACS in Digital Imaging and Communications in Medicine (DICOM) format. These files are loaded onto local servers for analysis. As imaging files are not amenable to the SQL format, files are linked by adding their accession numbers, file names, and download file paths to REINAH tables. Imaging characteristics determined from these scans are imported directly by the data team and linked by accession number and patient ID. Patient outcome data are stored in REDCap (Research Electronic Data Capture; Vanderbilt University) and imported in tabular form. The resulting database objects are primarily interconnected through generated registry-specific patient ID and encounter numbers, with patient images linked through PACS accession numbers. This structure provides data granularity at the encounter level, with the flexibility to be compiled into patient-level information for specific metrics. Data imports and assessments are refreshed weekly via scheduled SQL server–based ETL jobs, with oversight by team data engineers. The ETL process is summarized in Figure 1, with an overall flow sheet of the REINAH structure provided in Figure 2. This architecture is built with the capability to scale up to more volume and be repurposed for further downstream analytics. Data complexity is handled without compromising the performance efficiency of data operations. The 3 Rs—reliability, reusability, and reporting—were the driving goals of the system design.

#### Encounter Summaries

As ETL processes return interconnected big data sets, there is a need to aggregate information for quick reference and reporting. Encounter summary SQL objects are designed to provide core information regarding the patient during the primary encounter. This information includes the following: (1) primary identifiers for the patient and their encounter; (2) admission method and date and time; (3) discharge disposition, date and time, and binary in-hospital mortality; (4) patient address at the time of admission; (5) height, weight, and BMI; (6) Charlson Comorbidity Index (CCI) values with and without age score; (7) comorbidities included in the CCI score; (8) CCI-based life expectancy; (9) the number of stroke encounters relative to stroke type (eg, first, second, or third AIS encounters); and (10) patient demographics (age, gender, race, ethnicity, and marital status).

These are supplemented by measures of the state- and national-level Area Deprivation Index (ADI) [20-22] determined from the patient’s geographic address at the time of admission. The ADI provides a comprehensive assessment of the patients’ living conditions at the state and national levels, including measures of employment, property ownership, and utility access [20,22]. This summary forms a basis of patient information that may serve as a central nexus to connect a range of information that may be both collected within the EHR and generated by external sources.

#### Additional EHR Information

**Figure 3:**
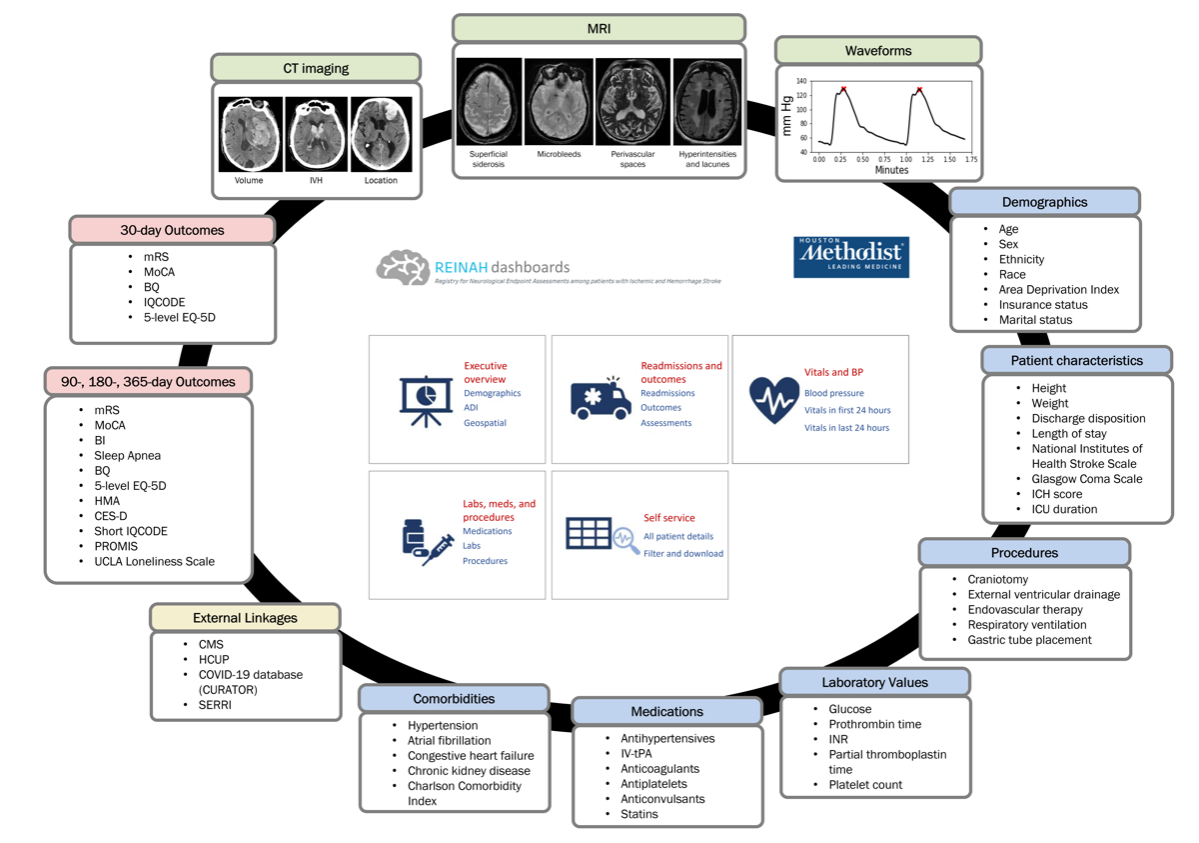
A summary of the different elements incorporated into the Registry of Neurological Endpoint Assessments among Patients with Ischemic and Hemorrhagic Stroke (REINAH) database. Primary data (blue) from hospital encounters are incorporated through the electronic health record, supplemented by additional measures of patient outcome (pink) and imaging (green) information obtained by the primary research team. Elements of the REINAH database also incorporate or extend to external resources both within Houston Methodist Hospital and at the state and national levels (yellow). BI: Barthel Index; BQ: Berlin questionnaire; CES-D: Center for Epidemiological Studies Depression Scale; CMS: Centers for Medicare and Medicaid Services; CT: computed tomography; CURATOR: COVID-19 Surveillance and Outcomes Registry; HCUP: Healthcare Cost and Utilization Project; HMA: Hill-Bone Medication Adherence Scale; ICH: intracerebral hemorrhage; ICU: intensive care unit; INR: international normalized ratio; IQCODE: Informant Questionnaire on Cognitive Decline in the Elderly; IVH: intraventricular hemorrhage; IV-tPA: intravenous tissue plasminogen activator; MoCA: Montreal Cognitive Assessment; MRI: magnetic resonance imaging; mRS: modified Rankin Scale; PROMIS: Patient-Reported Outcomes Measurement Information System; SERRI: Sepsis Early Recognition and Response Initiative; UCLA: University of California, Los Angeles.

The aforementioned encounter overviews are designed to provide a focal point of reference that can be connected with other data resources. Data residing within the EHR extend well beyond these stroke encounter overviews; however, an effective investigation of stroke conditions requires a comprehensive approach. As part of the aforementioned ETL process, data related to patient vitals, administered and historical medications, operations, ICU metrics, and laboratory values are separated into individual database objects that are linked to summaries through patient ID and encounter numbers. The data outline the care administered in both specific and generic terms, the time at which that care was ordered, the time of provision, and any applicable notes or results. Laboratory records include the dates and times of order and analysis, result values, and analysis units. Considering the breadth of the patient population, this creates a large amount of cleaned and assessable information; however, it does not encompass the entirety of potentially extractable relevant data. The flexible nature of the database architecture and the experience of the data team will allow the repository to grow and adjust as new information emerges regarding potential factors of import related to the occurrence and consequences of stroke. The overall contents of the REINAH database are summarized in Figure 3.

### Non-EHR Data Sources

#### Imaging Assessments

Patients who underwent a CT linked to a primary ICH encounter have the aspects of their hemorrhage recorded. Hemorrhage volume is assessed via the ABC/2 method [23], and records are created for the location of the hemorrhage (lobar, deep, brainstem, or cerebellum), laterality of the hemorrhage, and presence of any intraventricular hemorrhage. Additional notes are retained documenting the presence of additional simultaneous hemorrhage types (eg, subarachnoid or subdural) or present causes of hemorrhage (eg, metastases or ischemic transformation). In this way, the inclusion of patients in the ICH cohort is directly verified by the research team. In the event that the primary assessor finds irregularities or is uncertain about the imaging assessment, the images are marked for adjudication within the image assessment SQL object. The data research team then meets to review the particular image or feature of concern and decide on the final assessment. Systematic assessment in this manner also enables the determination of the overall ICH score [24], which is automatically calculated and included in the database.

**Figure 4:**
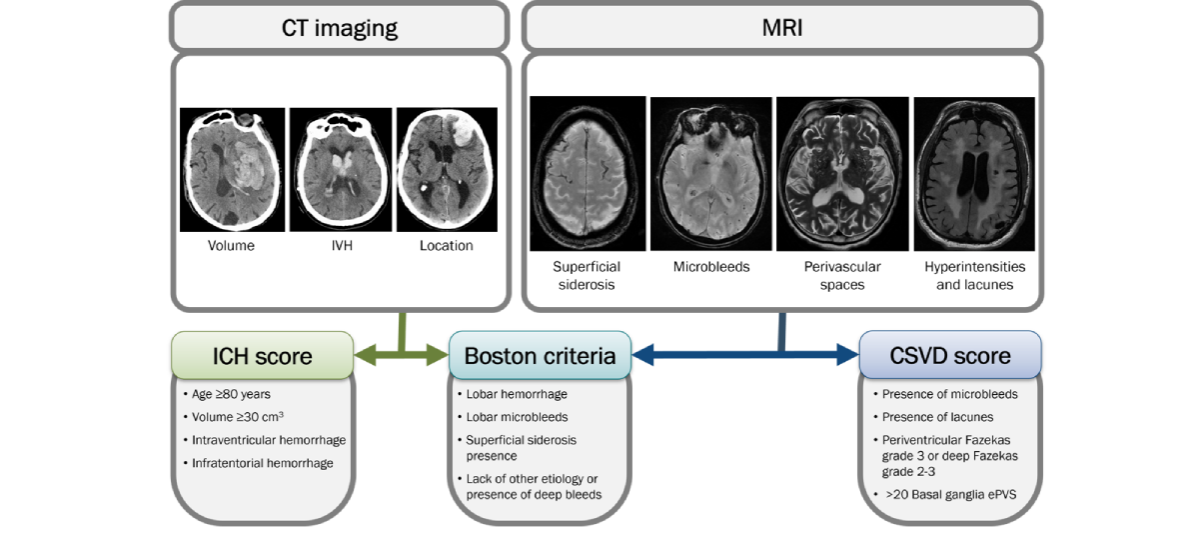
A summary of the elements recorded during imaging assessment and the calculation of the total cerebral small vessel disease score. CT: computed tomography; ePVS: enlarged perivascular spaces; ICH: intracerebral hemorrhage; IVH: intraventricular hemorrhage; MRI: magnetic resonance imaging.

In addition to the direct assessments of hemorrhage characteristics, MRI is used to assess for background lesions within patients’ brain parenchyma. As with the hemorrhage assessments, manual assessment of MRIs is performed by trained personnel. Primary features of interest include lesions indicative of small vessel disease or CAA. Gradings performed on each patient include the following: (1) Fazekas-scored periventricular and deep white matter hyperintensities, (2) the total number of cortical microbleeds, (3) the number of superficial cortical microbleeds, (4) the total number of lacunes, (5) the total number of sulci exhibiting cortical superficial siderosis, and (6) enlarged perivascular spaces graded in the basal ganglia and centrum semiovale (Figure 4) [25].

These measures allow for assessment using the modified Boston criteria [26] for CAA as well as the calculation of the total cerebral small vessel disease (CSVD) score [25], both of which were incorporated for automatic assessment as data are uploaded to the repository. The presence of accessible MRIs will allow for the verification of both hemorrhagic and ischemic stroke.

In addition to the primary stroke population, a control population of patients without stroke with available imaging assessments is included in REINAH. Patients with stroke are matched (age, sex, race and ethnicity, comorbidity burden, ADI, and encounter year of imaging) with imaging controls via a 1-to-3 propensity score–derived algorithm, which seeks to balance the major risk factors contributing to ICH etiologies between patients with and without ICH. This provides a more focused assessment of the specific factors that separate patients who had an ICH from those who did not. To ensure sufficient data availability, 3 matched controls are recorded for each patient with stroke, and the relevant imaging will undergo similar CSVD and CAA assessments.

#### Patient Outcome Assessment

Although EHR data allow for the evaluation of in-hospital outcomes after stroke, this access is limited. The availability of EHR-based outcomes inherently relies on information based on contacts with the HM system and may not accurately capture patient trajectories should they be lost to follow-up or deceased without further contact. To address this data gap, a customized phone registry for contacting and assessing patients with stroke was created within the larger REINAH project. Trained research staff reach out to patients 30, 90, 180, and 365 days after discharge from their stroke encounter and administer a custom battery of assessments designed to assess the cognitive and functional status of patients as well as their quality of life. The window for assessment varies based on the time point and assessment of interest. The windows for the 30-, 90-, 180-, and 365-day assessments, respectively, extend for 14, 28, 42, and 60 days after the focal time point is reached. Up to 5 call attempts may be made at each time point to provide adequate opportunity for patient contact, with callback numbers left after each unsuccessful attempt. Once contact is made, the contacting team member describes the purpose and process of outcome data collection, including the type of interview and time points of contact. Additional information and documentation are provided via email upon request. After patients affirm their understanding of the process, consent is obtained. This process is witnessed by a passive observer to ensure that proper procedures are followed and that valid consent is obtained. As no in-person contact is made with patients, consent documentation is signed by the contacting team member and witness and stored in REDCap servers.

The neurocognitive assessment battery used during these interviews is summarized in Table 1. Patient outcomes and end points recorded during this process are attributed to the intended contact time point. Patients who screened positive for depression during the outcome assessment are provided with mental health resources and urged to consult with their primary care physician.

This collective battery is intended to provide a comprehensive assessment of patient status and can generally be completed within 20 to 25 minutes of contact. To avoid possible language barriers, an interpretation service is enlisted during calls in which the patient is reported to have a non-English primary language. In this case, the translator will provide an additional signature on the consent documentation along with the contacting team member and witness. If patients are unable to directly participate, caregivers are also able to provide information regarding patient condition. Measured outcomes extend beyond the raw metrics of functional ability and include additional measures such as medication adherence and sleep apnea, which have a substantial impact on patient health and are often not captured by standard functional outcome assessments [27,28]. The obtained outcome measures are stored in REDCap with the affiliated consent documentation.

**Table 1:** Outcome assessments performed within the Registry of Neurological Endpoint Assessments among Patients with Ischemic and Hemorrhagic Stroke (REINAH) project.

Measurement	Assessment type	Assessment details
Modified Rankin Scale	Functional	Measures level of disability or death
Barthel Index	Functional	Measures the ability to perform activities of daily living
MoCA^a^	Cognitive	General cognitive assessment
UCLA^b^ Loneliness Scale	Quality of life	Assesses feelings of loneliness and social isolation
EQ-5D-5L	Quality of life	Assesses the ability to perform activities of daily life and quality of life
Berlin Questionnaire	Sleep apnea	Assessment of possible apnea and frequency
Hill-Bone Medication Adherence Scale	Medication adherence	Self-assessment of medication adherence
CES-D^c^	Quality of life	Assesses presence and frequency of depressive symptoms
IQCODE^d^	Cognitive	Proxy assessment of cognitive decline

^a^MoCA: Montreal Cognitive Assessment.

^b^UCLA: University of California, Los Angeles.

^c^CES-D: Center for Epidemiological Studies Depression Scale.

^d^IQCODE: Informant Questionnaire on Cognitive Decline in the Elderly.

### Data Harmonization

With the large amount of disparate data housed in REINAH, there is a need to control for variability and error within the imported EHR data and ensure that clean data elements are available for upfront reporting. A data harmonization effort was undertaken to provide clear output summaries. Data elements from each SQL object were reviewed by the data team, and customized data rules were implemented to reduce variability in record reports. Time formats, units, and text styles (eg, “right” vs “R”) were standardized and unified across tables, with consistent rules applied to each object. Conditions that may be disparate within the EHR but that are meaningfully different within a cerebrovascular context (eg, diabetes with complications vs diabetes without complications) are grouped for more cogent reporting. To avoid data loss, harmonized data are provided as additional “views” that draw from the ETL-imported SQL objects without altering them; the underlying raw data remain complete so that factors aggregated during harmonization may still be explored.

### Data Access

Summary access to REINAH data sets is provided through graphical dashboards that present as clickable web pages viewable using web browsers on the institutional intranet. These provide a broad range of cohort characteristics, with separate data presentations for demographics, common comorbidities, imaging profiles, and discharge characteristics. Dashboards are created using Tableau (Tableau Software, Inc), a visual data analytics tool designed to provide accessible summaries of complex raw data. Local investigators may use these dashboards to preview data availability and apply preset demographic filters to assess the feasibility of any studies of interest. After preview, they may contact the data team to provide flat data sets for analysis. Curated data sets are also used directly by the data science team for advanced analytics and research initiatives.

### Extensions to Other Systems

#### Extensions to Other HM Projects

##### Overview

The development of the REINAH resource was driven by the need for a comprehensive clinical resource to support detailed study of patients with stroke. In this spirit, REINAH has been constructed to interface with other data resources by retaining the patient and encounter identifiers from the EHR. Projects within HM may be linked using the patient MRN numbers, and currently linked resources include those outlined in the following sections.

##### COVID-19 Surveillance and Outcomes Registry

The health care environment tasked with caring for patients with stroke has faced large-scale disruption throughout the recent COVID-19 pandemic, and patients experiencing COVID-19 infection now present with an uncertain comorbidity with potentially onerous lingering effects. The COVID-19 Surveillance and Outcomes Registry (CURATOR) was developed at HM at the onset of the pandemic and has been tracking metrics of SARS-CoV-2 infection to support cross-sectional and longitudinal research [29]. Guided by the same drive toward LHS implementation and built upon similar data pipelines, CURATOR retains information for all patients tested for SARS-CoV-2 or administered a COVID-19 vaccine at any HM location. CURATOR’s design also allows for the capturing of longitudinal information through previous and subsequent patient encounters. Patients receiving direct patient care associated with COVID-19 hospitalization also have in-hospital procedures and metrics recorded for assessment. CURATOR and REINAH are built upon the same data structure, providing a direct, cohesive avenue for investigating the interplay between COVID-19 and stroke.

##### VICU Resource

The ICU environment is a critical aspect of patient care and recovery. Recognizing this, HM sought to enhance technological integration within the ICU environment as an aspect of its telecritical care program. This VICU is designed to provide live, accurate monitoring of patient condition, including heart rate, ECG, electroencephalogram, respiratory rate, blood pressure, blood oxygenation, ventilator settings, and intravenous fluid administration. Additional video monitoring was also implemented, with cameras in the patient rooms feeding to an operations center at the HM main location within the TMC. Extracted waveform data provide a unique opportunity to investigate time-sensitive aspects of poststroke care and recovery, generating actionable insights.

##### SERRI Resource

Infection has a dynamic relationship with stroke, recognized as both a cause and consequence of stroke events and a contributing factor to poor poststroke outcomes. Motivated to reduce sepsis-linked costs and mortality, HM established the SERRI to track and assess markers of infection, sepsis, and systemic inflammatory response syndrome. Patients within the ICU are monitored for these conditions every 12 hours by trained nurse practitioners to maintain accurate, time-sensitive tracking of patient condition.

##### Delirium

Similar to sepsis and infection, delirium is a relatively common neurocognitive consequence of stroke and ICU hospitalization that appears as an acute confusional state [30,31]. Although delirium portends cognitive impairment and poorer functional outcomes, it is often difficult to trace because of its transient nature. To improve this, HM protocol includes regular delirium assessments via the Confusion Assessment Method for the ICU [32] or modified Arousal, Attention, Abbreviated Mental Test–4, and acute change delirium assessment tools [33,34] for all patients aged >70 years and those within the ICU.

#### Links to External Projects

##### Overview

Extending beyond the research initiatives at HM, data housed within the REINAH repository can be cross-referenced with outside data sources. This will enable large-scale analyses and benchmarking of the REINAH stroke population. Institutional data use agreements were established for the linkage and ingestion of the projects outlined in the following sections.

##### CMS Data

Although these data will be specific to patients with Medicare and Medicaid as the payers for their hospital visits, the older age of patients with ICH and stroke will provide a large population with available payer information. This data source allows for the study of variables affecting patient outcomes, such as prescription and medication costs, the frequency and consistency of prescription fulfillment, and additional medical provision beyond the HM system. This will provide a detailed cohort that can more reasonably be assessed for adherence and direct financial burdens.

##### Healthcare Cost and Utilization Project

Available through the Agency for Healthcare Research and Quality, Healthcare Cost and Utilization Project data provide longitudinal information regarding hospital care and expenses designed to support hospital policy research. This includes a number of specialized databases focused on pediatric, inpatient, surgical, and readmission events at both the state and national levels. This can be used to provide greater local and national context for patients within the REINAH repository.

##### ADI Data

Provided by the Center for Health Disparities Research at the University of Wisconsin School of Medicine and Public Health, the ADI, as previously described, provides an aggregate measure of socioeconomic deprivation faced by patients. The ADI aggregates information regarding employment, education, resource ownership and availability, and living conditions presented in deciles at the state level and percentiles at the national level. The patient addresses provided at each encounter are geocoded to determine both state and national ADI values, which are incorporated directly into REINAH encounter overviews.

### Regulatory Processes and Governance

#### Overview

The REINAH protocol and all its constituent processes are maintained under the governance of the HM institutional review board (IRB). This review includes all aspects of data collection, assessment, patient contact, image grading, and external data incorporation. The governing protocol is maintained by project management staff within the HM Department of Neurosurgery. All data maintained within REINAH are stored on encrypted, password-protected servers and devices. Protocols and procedures are subject to annual review by the IRB in concert with governance from the institutional leadership team. All patient contacts during the outcome assessment collection process are witnessed by a secondary team member to ensure that proper procedures and conduct are followed. Projects seeking to use REINAH as a primary data source undergo independent IRB review, approval, and audit. Additional transfer agreements are required and maintained for data released to collaborators. Any released data are stripped of identifiers before release, and any racial, ethnic, or clinical categories with few enough data points to be considered identifiable are recoded or redacted (in cases where recoding is not possible).

#### Support Team

To support the breadth of the REINAH platform, a diverse and experienced team is necessary. Dedicated data analysts and infrastructure architects are needed to carry out the ETL processes, maintain registries, and provide dashboard support. Data scientists and researchers with imaging experience are required to manage imaging imports, provide assessments of intracranial patient characteristics from CT and MRI scans, and ensure data quality. Similarly, clinical researchers and epidemiologists are needed to orient the database and ensure that clinically relevant and meaningful data are made available. Administrators, managers, and coordinators support safe, effective operations within the hospital environment and guide extensions to clinical trials. Finally, a team of research assistants and technicians conduct patient outcome assessments and effective research both within and across hospital departments.

### Ethics Approval, Informed Consent, and Participation

The REINAH system operates with ethical review and approval from the Houston Methodist Institutional Review Board to ensure that all data are secured and appropriately used (IRB ID: MOD00005579). Informed consent is obtained from eligible patients or, in cases in which patients are unable to consent, legally authorized representatives, with consent allowing for secondary analysis and disclosure of health data. Data withdrawn from REINAH are stripped of direct identifiers to protect the confidentiality and privacy of patients, and data sets generated for specific projects are recoded using REINAH-specific patient ID numbers to prevent data from becoming identifiable via a small group. Patients participating in outcome assessments receive no direct compensation, and the only benefits conferred are through the use of data to enhance clinical knowledge and advance patient care.

### Statistics

Patient demographics and characteristics are presented for the overall cohort and for the AIS, ICH, TIA, and SAH subcohorts. Numerical factors are presented as medians and IQRs, with categorical classifications presented as proportions.

## Results

### Resulting Database Structure and Access

**Figure 5:**
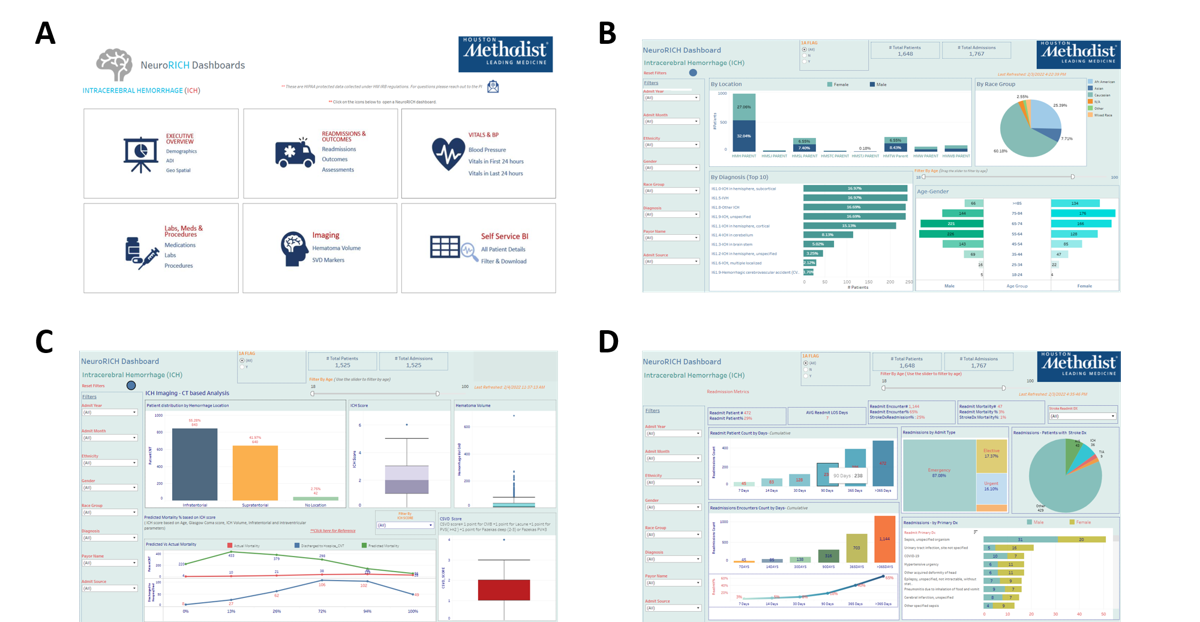
Examples of dashboards provided for data exploration. (A) The top-level welcome dashboard; (B) intracerebral hemorrhage (ICH) summary dashboard, including age and race distributions, diagnosis codes, and admission hospitals; (C) dashboard summary of imaging factors for patients with ICH, including hemorrhage assessments, cerebral small vessel disease score, ICH score, and subsequently predicted mortality; and (D) readmission summary dashboard for patients with ICH, including readmission type, diagnoses, and readmission time. All dashboards are filterable to improve access.

A large amount of information is housed in the outlined database. At present, the REINAH database covers 463 GB, with imaging data obtained for patients with ICH spanning >2 TB. Weekly ETL processes to refresh the database take approximately 3 hours, with an additional 30 minutes, approximately, required to perform geocoding processes for ADI calculation. Initial data incorporation split the data into SQL tables for patient information, encounter information, comorbidities and initial dates of record, administered and outpatient medications, flow sheet data, admission diagnoses, discharge diagnoses, outcomes, procedures, and imaging records. Using these raw import tables, summary tables were constructed for each specific subtype of interest. Laboratory values, clinical scores, and flow sheet data characteristics for the first 6 hours, first 24 hours, and last 24 hours were preallocated for quick reference. Collected patient outcome information is imported directly from REDCap, whereas imaging assessments are imported as CSV files generated by the grading and import processes. All tables are interlinked using the patient ID, encounter number, or imaging accession number to create a fully interlinked structure. Front-end dashboards providing summary access were generated, with example dashboards for the ICH cohort shown in Figure 5. At welcome, users may navigate to the module most relevant to their query and begin exploring REINAH data using the provided interfaces and live filters. Data can be requested for download through this dashboard for provision to IRB-approved research projects.

### Overall Cohort and Data Availability

As of June 2022, the REINAH registry contains 18,061 patients with primary stroke encounters at HM. Of these 18,061 patients, 1809 (10.02%) recorded a primary ICH encounter, 13,444 (74.43%) recorded a primary AIS encounter, 3165 (17.52%) recorded a primary TIA encounter, and 1221 (6.76%) recorded a primary SAH encounter. The overall cohort is 48.35% (8732/18,061) male and 51.65% (9329/18,061) female. Ethnic information, stored separately from race, showed that 15.65% (2826/18,061) of the population identified as Hispanic. Racial information is available for 96.82% (17,487/18,061) of the patients, with the patient cohort including 0.67% (118/17,487) American Indian, 5.64% (986/17,487) Asian, 25.41% (4444/17,487) Black, 0.2% (35/17,487) Hawaiian or Pacific Islander, 66.38% (11,608/17,487) White, and 1.69% (296/17,487) other patients. The median patient age at the initial encounter was 71 (IQR 60-80) years. The median National Institutes of Health Stroke Scale scores for patients with ICH and AIS within the database are 9 (IQR 2-21) and 3 (IQR 1-7), respectively. Median Glasgow Coma Scale scores for patients with ICH and AIS are 13.5 (IQR 7-15) and 15 (14-15), respectively. Individual cohort demographics and characteristics are provided in Table 2.

**Table 2:** Demographics and discharge characteristics for Registry of Neurological Endpoint Assessments among Patients with Ischemic and Hemorrhagic Stroke (REINAH) cohorts.

			AIS^a^ (n=13,444)	ICH^b^ (n=1809)	TIA^c^ (n=3165)	SAH^d^ (n=1221)
**Demographics**						
	Age (years), median (IQR)		69 (59-79)	67 (55-78)	71 (60-80)	65 (53-76)
	Female sex, n (%)		6831 (50.8)	836 (46.2)	1767 (55.8)	736 (60.3)
	**Race, n (%)**					
		Asian	760 (5.7)	152 (8.4)	104 (3.3)	86 (7)
		Black	3536 (26.3)	428 (23.7)	676 (21.4)	213 (17.4)
		White	8733 (65)	1157 (64)	2308 (72.9)	867 (71)
		Other	415 (3.1)	72 (4)	77 (2.4)	55 (4.5)
	Hispanic, n (%)		2018 (15)	381 (21.1)	427 (13.5)	264 (21.6)
	Top 30% ADI^e^, n (%)		2528 (18.8)	354 (19.6)	538 (17)	154 (12.6)
**Comorbidities**						
	Hypertension, n (%)		10,629 (79.1)	1456 (80.5)	2551 (80.6)	839 (68.7)
	Diabetes, n (%)		7165 (53.3)	708 (39.1)	1370 (43.3)	282 (23.1)
	Atrial fibrillation, n (%)		7551 (56.2)	460 (25.4)	670 (21.2)	152 (12.4)
	Chronic kidney disease, n (%)		4750 (35.3)	607 (33.6)	713 (22.5)	163 (13.3)
	Congestive heart failure, n (%)		4651 (34.6)	476 (26.3)	663 (20.9)	159 (13)
	CCI^f^, median (IQR)		5 (3-8)	6 (3-9)	4 (2-6)	2 (0-4)
**Medication history, n (%)**						
	Antihypertensives		4669 (34.7)	557 (30.8)	1203 (38)	397 (32.5)
	Antiplatelet		4617 (34.3)	454 (25.1)	1232 (38.9)	384 (31.4)
	Anticoagulants (any)		2153 (16)	274 (15.1)	577 (18.2)	197 (16.1)
	Statin		4847 (36.1)	533 (29.5)	1289 (40.7)	419 (34.3)
	Warfarin		497 (3.7)	73 (4)	144 (4.5)	51 (4.2)
	Novel oral anticoagulants		1525 (11.3)	181 (10)	401 (12.7)	98 (8)
**Imaging**						
	CT^g^ availability, n (%)		12,827 (95.4)	1780 (98.4)	2989 (94.4)	1048 (85.8)
	Hemorrhage volume, median (IQR)		N/A^h^	10.8 (2.9-32.5)	N/A	—^i^
	MRI^j^ availability, n (%)		11,500 (85.5)	1265 (69.9)	2658 (84)	340 (27.8)
	CSVD^k^ score, median (IQR)		—	2 (1-3)	—	—
**Outcomes**						
	Length of stay, median (IQR)		4 (2-6)	6 (3-13)	2 (2-4)	2 (0-8)
	**Discharge disposition, n (%)**					
		Home	7967 (59.3)	553 (30.6)	2641 (83.4)	391 (32)
		Deceased or hospice	899 (6.7)	484 (26.8)	25 (0.8)	104 (8.5)
		Skilled nursing facility	1388 (10.3)	207 (11.4)	205 (6.5)	63 (5.2)
		Long-term acute care	266 (2)	191 (10.6)	9 (0.3)	46 (3.8)
		Rehabilitative care	2588 (19.3)	346 (19.1)	214 (6.8)	84 (6.9)
		Other or transfer	336 (2.5)	28 (1.5)	71 (2.2)	533 (43.7)
	30 days, n (%)		—	53 (2.9)	—	—
	90 days, n (%)		—	72 (4)	—	—
	180 days, n (%)		—	69 (3.8)	—	—
	365 days, n (%)		—	76 (4.2)	—	—

^a^AIS: acute ischemic stroke.

^b^ICH: intracerebral hemorrhage.

^c^TIA: transient ischemic attack.

^d^SAH: subarachnoid hemorrhage.

^e^ADI: Area Deprivation Index.

^f^CCI: Charlson Comorbidity Index.

^g^CT: computed tomography.

^h^N/A: not applicable.

^i^Not available.

^j^MRI: magnetic resonance imaging.

^k^CSVD: cerebral small vessel disease.

### Imaging

Imaging assessments at the time of writing were performed manually. As the REINAH project grew out of an initial repository focused on ICH, this patient population has received the primary effort of image analysis and assessment. Assessable CT and MRI scans were available for 98.4% (1780/1809) and 69.9% (1264/1809) of patients with ICH, respectively, and were completed to this level. The median hemorrhage volume was 10.8 (IQR 2.9-32.5) cm^3^. Identified hemorrhages were also tracked for location and the presence of intraventricular hemorrhage; 43% (765/1780) of hemorrhages were found in lobar regions, 41.91% (746/1780) were found in deep brain regions, 10.67% (190/1780) were found in the cerebellum, and 4.44% (79/1780) were found in the brainstem. Intraventricular hemorrhage was identified in 36.91% (657/1780) of patients with ICH.

MRIs were available for approximately 69.87% (1264/1809) of cases. Patients assessed for CSVD score showed a median CSVD score of 2 (IQR 1-3). In total, 16.8% (212/1264) of patients with ICH were found to have a score of 0, a total of 28.8% (364/1264) had a score of 1, a total of 27.3% (345/1264) had a score of 2, a total of 18.5% (234/1264) had a score of 3, and 8.6% (109/1264) had a score of 4. Following the modified Boston criteria assessment based on these same assessments, 76.58% (968/1264) of hemorrhages were not CAA related, 18.43% (233/1264) were rated as possible CAA, and 4.98% (63/1264) were rated as probable CAA.

### Outcome Measurement

In ICH follow-up outcome measurements, 428 total patients became eligible for enrollment. Of these 428 patients, 240 (56.1%) responded. Of the responders, 71.3% (171/240) provided consent for assessment, and 28.8% (69/240) declined to participate. Functional outcomes were recorded for 31% (53/171) of patients at the 30-day time point (modified Rankin Scale [mRS] score median 3, IQR 1-4), 42.1% (72/171) of patients at the 90-day time point (mRS score median 2.5, IQR 1-4), 40.4% (69/171) of patients at 6 months (mRS score median 3, IQR 1-4), and 44.4% (76/171) of patients at 1 year (mRS score median 3, IQR 2-4).

## Discussion

### Principal Findings

Arising through the dedicated efforts of a diverse team, the REINAH repository has been established to support effective stroke research. An ETL process was first constructed to transform administrative EHR data into a patient- and encounter-focused format that supports cross-sectional and longitudinal investigations. This was also supported by imaging and outcome assessment pipelines that were developed to provide high-quality, detailed information on patient condition and long-term outcomes after discharge, well beyond that typically collected during a hospital encounter. The resulting data structure houses data from >18,000 patients with primary CVD and stroke encounters across CVD and stroke subtypes. Relevant imaging for patients was imported into local servers, and the hemorrhage and CSVD characteristics of patients with ICH were assessed. Data imported to REINAH are split into categorized SQL objects that can be rapidly arranged to produce the flat data sets necessary for focused investigations. Data from the EHR and PACS are supplemented by the prospective assessment of patient outcomes up to a year after discharge and data withdrawn from free-text notes via natural language processing. Finally, front-end access is supplied by a series of dashboards that can be used to investigate data before formal data requests are made. This concerted effort has yielded a research database that is comprehensive and accessible and stands to accelerate stroke research.

### Comparisons With Previous Systems

Knowledge and development of effective LHSs have accelerated over the past decade. However, systems have primarily focused on oncology, cardiology, and neurology, with little direct implementation within the domains of CVD and stroke. In total, 2 resources that have been developed are the Stroke Neuroimaging Phenotype Repository and the American Heart Association Get With The Guidelines (GWTG)–Stroke Registry [35,36]. These resources have notable impacts of their own but with differently focused purposes—the Stroke Neuroimaging Phenotype Repository is a specific imaging-focused database that does not provide the bulk of patient data or the longitudinal patient timeline captured by the REINAH data pipelines [35]. In contrast, GWTG provides a large-scale national infrastructure in which participating institutions voluntarily provide patient treatment and outcome metrics [36]. This breadth equips GWTG as a foundational piece to investigate large-scale treatment metrics and national disparities but forgoes the granularity, precise data definitions, and patient-centered focus that are achievable at local levels and necessary to drive institutional LHS progress. The REINAH structure, created in a similar structure to that of the CURATOR database [29], is designed to provide rapidly synthesizable, focused insights into the factors that lead to CVD onset and outcomes. The diverse pipelines outlined in this paper provide several advantages. First, owing to the size of both the local population and HM hospitals, drawing from the EHR provides a large, diverse patient cohort for assessment. Included patient numbers are comparable with those of major AIS and ICH clinical trials [37,38], providing a considerable platform for large-scale research initiatives. Supplementing direct EHR data with pipelines that capture unstructured data from imaging and outcomes creates a robust data environment that can be validated across data streams (eg, diagnoses can be reinforced by imaging analyses, whereas imaging findings may be supported by clinician notes) and enables randomized clinical trial–style observational projects.

Governance and ethical issues in LHS implementations were recognized, stemming from the need to balance patient safety and security with the benefits conferred by a focused study of their data. The REINAH structure and governance largely mitigate these concerns by operating under a dedicated IRB with regular review, implementing patient IDs specific to the research structure, restricting direct access to the data and encoded data structures, requiring subsequent studies to complete independent IRB reviews, and collecting witnessed consent for prospective outcome collection. The constructed dashboards provide an additional level of safety as investigators may preview and explore available REINAH data without the risk of exposing sensitive data. At the same time, providing this data perspective reduces the amount of time needed to conceptualize and return mature research data sets. Following these processes, REINAH-based research projects have been published and presented at international conferences to inform our future efforts in treating CVD [39-44].

### Limitations

Although the current iteration of REINAH provides a rich foundation to support stroke research, the system has some limitations and areas for development. First, REINAH represents a focused, institutional resource for CVD research, which limits the full investigation of nationwide or regional trends and restricts data to the current implementation of the EHR. This introduces the possibility of both geographical and temporal bias within the data set. In contrast, the institutional focus of the REINAH database allows for more detailed, consistent data definitions across a range of specialized topics that would be challenging or infeasible to implement on a larger scale and established connections with larger nationwide resources, allowing the REINAH cohort to be benchmarked within the larger scope of CVD research. Historical data from the HM system are also available, although they currently exist in a different EHR format and will require additional ETL development to be cohesively incorporated into the REINAH environment. In addition, imaging assessments currently require time-intensive manual grading for patients with ICH and AIS. Machine learning algorithms are being implemented to support the automated extraction of hemorrhage, stroke, and CSVD characteristics for all patients with available imaging. Similarly, although ECG and blood pressure waveforms are available and linked to patient encounters, summarized data characteristics are not yet available for analysis. Although current patients are highly characterized, records can be expanded by including caregiver or household contact information to gain insight into patient social support. This remains a prospective topic for future consideration; however, outreach may be supported by mobile apps and wearable devices, which are owned by up to 85% of the adult US population. Finally, data storage and analysis structures can migrate to cloud platforms to provide more flexible storage structures, and a formal data request application with automated SQL query construction is being incorporated into the front-end dashboards to improve access and further reduce downtime. Direct linkage with imaging is also being established to provide clickable links for viewing anonymized imaging from CVD encounters.

Established as a comprehensive stroke registry with the potential to grow in capability, REINAH stands as a state-of-the-art resource that houses a multitude of cleaned, research-quality stroke data. The depth and breadth of this platform has been established to provide rapid, detailed investigation of critical patient populations. Through this, we hope to foundationally support research projects to drive advances in CVD knowledge and care, develop predictive models for patient outcomes, lay the groundwork for future randomized clinical trials, and reduce the burden of stroke on patients and providers alike.

## Abbreviations

ADI: Area Deprivation Index
AIS: acute ischemic stroke
CAA: cerebral amyloid angiopathy
CCI: Charlson Comorbidity Index
CMS: Centers for Medicare and Medicaid Services
CSVD: cerebral small vessel disease
CT: computed tomography
CURATOR: COVID-19 Surveillance and Outcomes Registry
CVD: cerebrovascular disease
DICOM: Digital Imaging and Communications in Medicine
ECG: electrocardiogram
EHR: electronic health record
ETL: extract, transform, and load
GWTG: Get With The Guidelines
HM: Houston Methodist
HMSA: Houston Metropolitan Statistical Area
ICD-10: International Classification of Diseases, 10th Revision
ICH: intracerebral hemorrhage
ICU: intensive care unit
IRB: institutional review board
LHS: learning health care system
MRI: magnetic resonance imaging
MRN: medical record number
mRS: modified Rankin Scale
PACS: picture archiving and communication system
REDCap: Research Electronic Data Capture
REINAH: Registry of Neurological Endpoint Assessments among Patients with Ischemic and Hemorrhagic Stroke
SAH: subarachnoid hemorrhage
SERRI: Sepsis Early Recognition and Response Initiative
TIA: transient ischemic attack
TMC: Texas Medical Center
VICU: virtual intensive care unit

## Data Availability

The Registry of Neurological Endpoint Assessments among Patients with Ischemic and Hemorrhagic Stroke (REINAH) database represents an institutional resource that contains potentially identifiable patient information, and the depth and breadth of the platform makes dissemination infeasible. As such, data from the platform are not broadly available. Specific requests regarding system structure data or metadata may be made to enable the dissemination of the model.
